# A Survey on the Organization and Operation of Level II/III Neonatal Intensive Care Units in Greece: A Comparison Between 2004 and 2022

**DOI:** 10.3390/children12010085

**Published:** 2025-01-13

**Authors:** Kosmas Sarafidis, Nicoletta Iacovidou, Eleftheria Hatzidaki, Ilias Chatziioannidis, Gabriel Dimitriou

**Affiliations:** 1 1st Department of Neonatology and Neonatal Intensive Care, Faculty of Medicine, School of Health Sciences, Aristotle University of Thessaloniki, Ippokrateion General Hospital, 54642 Thessaloniki, Greece; ihatzi@auth.gr; 2 Neonatal Department, National and Kapodistrian University of Athens, Aretaeio Hospital, 11528 Athens, Greece; niakobid@med.uoa.gr; 3 Department of Neonatology & NICU, Medical School, University of Crete, University Hospital of Heraklion, 71003 Heraklion, Greece; el.hatzidaki@uoc.gr; 4 Department of Pediatrics, Patras Medical School, University of Patras, University Hospital of Patras, 26504 Patras, Greece; gdim@upatras.gr

**Keywords:** neonate, intensive care, level of care, health policy, neonatal standards

## Abstract

Background/Objectives: Limited data exist on the organization and operation of Level II/III Neonatal Intensive Care Units (NICUs) in Greece; this retrospective cross-sectional survey explored their structure and functioning in 2004 and 2022. Methods: A structured questionnaire was utilized, along with demographic and perinatal data obtained from the Hellenic Statistical Authority. Results: Between 2004 and 2022, live births decreased by 28%, while the prematurity rate rose from 6.96% to 11.87% (*p* < 0.001). Significant regional differences were observed in the number of NICUs (*p* = 0.033), live births (*p* < 0.001), and NICUs per 10,000 live births (*p* = 0.025). In this survey, data from 20 Level III NICUs in 2004 and 22 NICUs (one Level II) in 2022 were analyzed. NICU admissions increased by 16.1% (*p* = 0.389), while the rate of admitted neonates/1000 live births increased from 13.5 to 21.8 (*p* < 0.001). In 2022, premature infants constituted 40.2% of NICU admissions. The number of board-certified neonatologists increased by 21.8% between 2004 and 2022 (*p* = 0.795), along with a rise in the ratio of neonatologists per 10,000 live births (from 14.8 to 25, respectively, *p* < 0.001). Conversely, there was a significant 17.2% reduction in the nursing staff by 2022 (*p* = 0.034). The number of available NICU beds also increased during the study period. The ratio of ventilators to intensive care beds significantly improved (*p* < 0.001). In 2022, new treatment modalities, like therapeutic hypothermia, were introduced, and most NICUs reported offering long-term follow-up programs. Conclusions: This survey highlights significant advancements in Level II/III NICU infrastructure and care capabilities, while emphasizing demographic changes and a critical shortage of neonatal nursing staff. These factors should be carefully considered by health authorities in the development of future neonatal care strategic planning in the country.

## 1. Introduction

Perinatal and neonatal care have evolved significantly over recent decades, driven by advances in technology, new treatment approaches, the establishment of perinatal centers, regionalized networks, and quality improvement initiatives [1,2,3,4]. Despite significant advancements, data on the organization and operation of Neonatal Intensive Care Units (NICUs) in Greece—a country with a population of 10.43 million (2022)—remain limited and primarily reflect earlier periods, which are less representative of modern neonatology [5,6,7]. In addition, the country lacks a centralized recording system for NICUs. As a result, critical aspects regarding their organization and operation are unknown, making it challenging to establish guidelines and implement evidence-based decision-making measures.

In the absence of high-quality neonatal databases, which are considered essential for assessing and improving neonatal care [8], conducting relevant surveys plays an important role in achieving this goal. Obtaining data related to NICUs, particularly regarding the number of neonates, acuity, staffing levels, and especially nursing staff, is highly valuable for identifying shortages, benchmarking against international standards, and taking steps toward a more nationally consistent, guideline-based approach to perinatal and neonatal care [9,10].

Recognizing the importance of this issue, the Board of the Hellenic Neonatal Society during the 2021–2023 period undertook a significant initiative to collect updated and comprehensive data on NICU organization and operation as a reflection of the state of perinatal–neonatal care in Greece. This effort aimed to provide a contemporary snapshot of NICU structure and functioning, allowing for meaningful comparisons with previous, unpublished data collected in 2004. By examining trends and changes over time, the survey seeks to identify persistent gaps, emerging challenges, and opportunities for improvement. This comparison is particularly important for evaluating the degree of regionalization, the efficiency of resource utilization, and the alignment of Greek NICUs with international best practices. Furthermore, this investigation could serve as a critical step toward establishing a foundation for continuous monitoring and quality improvement. Findings are expected to guide evidence-based policy development, support the implementation of targeted interventions, and ultimately contribute to improved neonatal outcomes nationwide, as highlighted by other relative studies [11].

## 2. Material and Methods

### 2.1. Study Design

This study is a retrospective cross-sectional survey, focusing on the years 2004 and 2022 and aiming to investigate the following: (1) Level II/III NICU regional distribution and characteristics, (2) admission rates, (3) human resources (medical and nursing staff), (4) facilities and technological equipment, (5) therapeutic interventions and access to specialized treatments, (6) neonatal follow-up programs, and (7) NICU affiliations and services.

### 2.2. Methodology

This survey utilized a structured questionnaire initially developed in 2005 (reflecting data from 2004) and revised in 2023 (reflecting data from 2022). To enhance the revised questionnaire and address any potential ambiguities, a virtual meeting was held in December 2022 with the directors of all Level II and III NICUs nationwide. Following its finalization, the questionnaire was distributed in early 2023 to the directors of Level II and III NICUs, as was done in 2005. In both time periods, directors received an invitation outlining the survey’s objectives and assuring the anonymity of the collected data. In 2005, the questionnaire was distributed in Word format, with the responses submitted as written documents. In 2023, however, the questionnaire was administered electronically via Google Forms, and the responses were subsequently exported to an Excel sheet for the analysis. The 2023 questionnaire (provided as Supplementary Materials File S1) consisted of 145 questions covering the seven previously mentioned domains. Frequent reminders were sent to non-responders until the 2022 data collection concluded in May 2023.

For the purposes of this survey, demographic–perinatal data, including the absolute numbers of live births, premature infants, low- and very-low-birth-weight (LBW-VLBW) infants, and prematurity rates were also obtained from the Hellenic Statistical Authority (ELSTAT) [12], both at the national and regional level. ELSTAT provides pregnancy duration data categorized by birth weight, divided into 500 g intervals, ranging from under 501 g to over 5001 g (e.g., <501 g, 501–1000 g, 1001–1500 g, etc.).

To evaluate the distribution and capacity of NICUs within the population, several indicators were calculated: (a) NICUs per 10,000 live births; (b) NICU beds per 10,000 live births; (c) NICU admissions per live birth; (d) neonatologists per NICU bed, as well as per 10,000 live births, 1000 live births, and per 1000 LBW-VLBW infants; and (e) nurses/midwives per NICU bed, as well as per 10,000 live births.

The American Academy of Pediatrics standards were applied to designate the NICU levels based on the types of care, services, and therapies provided [9,13,14].

## 3. Statistics

Descriptive statistics were used to analyze the survey results. The data were presented as counts and proportions for qualitative variables (e.g., NICU level, NICU sector) and as medians with ranges for quantitative variables (e.g., total admissions, LBW admissions), as these did not follow a normal distribution according to the Kolmogorov–Smirnov test. Comparisons were conducted using the Chi-square test and, when required, the Fisher’s exact test. Differences in proportions were evaluated using the z-test for proportions, while comparisons of arithmetic data were made using the Mann–Whitney U test, as appropriate. Furthermore, correlations were tested via the Spearman correlation coefficient (r_s_). The IBM SPSS software (version 23) and the R programming language (version 4.4.0) were used for the data analysis, with the significance level set at <0.05.

## 4. Results

### 4.1. National Demographic Perinatal Characteristics

According to the Hellenic Statistical Authority, live births in Greece declined by 28%, from 105,444 in 2004 to 75,921 in 2022. However, the number of premature neonates increased by 22.7% (from 7335 to 9009) during this period, with the prematurity rate rising by 70.4%, from 6.96% in 2004 to 11.87% in 2022 (*p* < 0.0001).

### 4.2. NICU Regional Distribution and Characteristics

**Figure 1:**
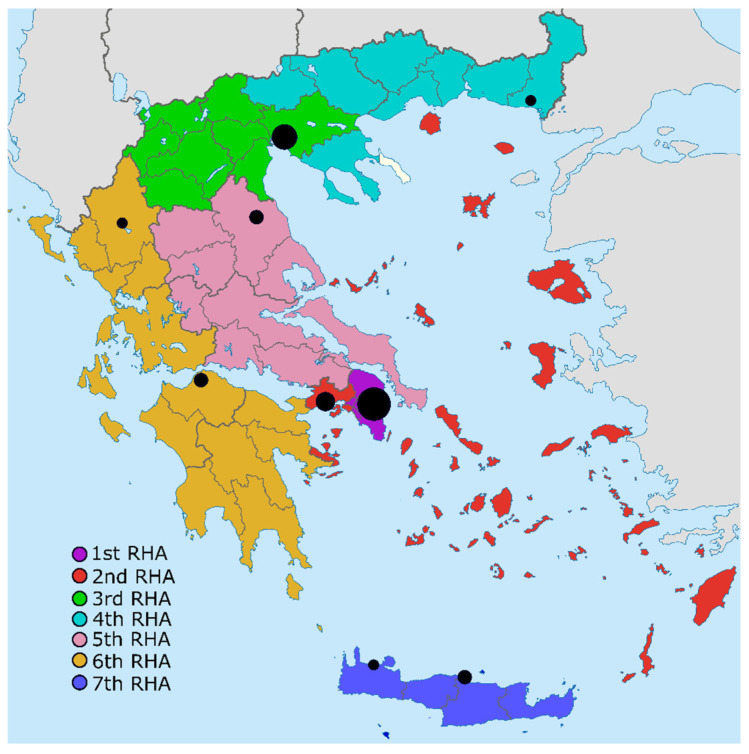
Regional Health Authorities (RHAs) and the distribution of Level II/III NICUs across Greece in 2022, with the cycle volume reflecting the number of NICUs in each city.

In 2022, Greece had 24 Level III NICUs and 3 Level II NICUs, compared to 20 Level III NICUs and 2 Level II NICUs in 2004, reflecting a 22.7% increase in the total number of Level II/III NICUs (from 22 to 27). In 2004, 17 NICUs operated in the public sector and 3 in the private sector, while in 2022, 20 out of 27 NICUs (74.1%) operated in the public sector. Furthermore, the number of university-based units has increased by 50% (from six to nine) since 2004. Figure 1 illustrates the distribution of Level II/III NICUs across the seven Regional Health Authorities, while Table 1 presents their distribution in relation to live births, prematurity rates, and the number of NICUs in 2022.

Significant regional differences were observed in the number of NICUs (*p* = 0.033), live births (*p* < 0.001), and NICUs per 10,000 live births (*p* = 0.025). In contrast, no regional differences were observed in the prematurity rate (*p* = 0.989) or the number of Level II/III NICUs (*p* = 0.132) (Table 1). Notably, Attica and Piraeus accounted for 37% and 5.95% of Greece’s total births, respectively, with Attica (the geographical region including Athens) recording the highest prematurity rate nationwide, at 13.4%. In relation to the national annual live births, there were 2.1 and 3.16 Level III NICUs per 10,000 live births in 2004 and 2022, respectively. However, this increase was not statistically significant (*p* = 0.156).

In this survey, all 20 of the existing Level III NICUs participated in 2004, whereas in 2022, 21 out of 24 Level III NICUs (87.5%) and 1 out of 3 Level II NICUs participated, resulting in a total response rate of 81.5%. In the 2004 registry, 17 NICUs operated in the public sector, 3 in the private sector, and 6 were affiliated with universities. By 2022, among the NICUs participating in the registry, 16 operated in the public sector, 6 in the private sector, and 7 were affiliated with universities.

### 4.3. NICU Admissions

Survey data indicate that NICU admissions rose by 16.1% from 2004 to 2022, increasing from 14,237 (median 504, range 226–2619) to 16,528 (median 380.5, range 204–3140) admissions (*p* = 0.389). During the same period, the rate of admitted neonates per live birth (calculated based on the annual national number of live births) rose significantly, increasing from 13.5 to 21.8 per 1000 live births, representing a 61.4% increase (*p* < 0.001).

According to the questionnaires, 46.3% of NICU admissions in 2004 involved neonates weighing less than 2.5 kg, a significant difference compared to the 32.6% reported in the 2022 survey (*p* < 0.001). Similarly, 9% of admissions in 2004 involved neonates weighing less than 1.5 kg, compared to 5.5% in 2022, though this difference was not statistically significant (*p* = 0.089). Data for neonates weighing less than 1 kg were only available for 2022, accounting for 1.8% of admissions involving Level III NICUs.

Data related to gestational age at birth and the care for extremely preterm infants born at less than 28 weeks were also available from 21 NICUs in 2022. More specifically, premature neonates under 37 weeks accounted for 40.2% of NICU admissions. Additionally, 9.9% of admissions involved neonates born before 32 weeks of gestation, while 2.1% were for those born before 28 weeks.

In 2022, NICU-supported hospitals recorded 41,910 births (median 948, ranging from 385 to 11,165 per hospital), based on data from 19 NICUs. Additionally, the vast majority (90.3%) of premature infants under 32 weeks (801 out of 887) were inborn; that is, they were born in hospitals equipped with NICU facilities (data from 17 NICUs). In 2004, in 13 out of 20 NICUs admitting both inborn and outborn neonates, 58.6% of the admitted neonates were inborn. However, in 2004, inborn infants were not recorded by gestational age categories (e.g., those born under 32 weeks).

### 4.4. Human Resources

#### 4.4.1. Medical Staff

The total number of board-certified neonatologists working in 22 Level II/III NICUs in 2022 was 190 (a median of 8 per NICU, ranging from 4 to 19), reflecting a 21.8% increase from 2004, when 156 neonatologists (median 8, range 4–12) served in 20 NICUs (*p* = 0.795). Additionally, 11 NICUs employed 16 pediatricians (median 1, range 0–3), a rise from 7 pediatricians across 2 NICUs in 2004. A positive correlation was observed in 2022 between the number of certified neonatologists and the number of intensive care beds in each center (r_s_ = 0.694, *p* < 0.001). However, no significant correlation was found between the number of neonatologists and NICU admissions during the same year (r_s_ = 0.172, *p* = 0.444).

The 2022 registry provided data on medical training. Eighteen Level III NICUs reported training 38 neonatal subspecialty fellows (median 2, range 0–4) over 24 months and 44 pediatric residents (median 2, range 0–7) over 6 months. In 2004, training included a median of four (range 1–4) neonatal subspecialty fellows in 12 NICUs and a median of two (range 1–5) pediatric residents in 11 NICUs.

Between 2017 and 2021, public NICUs trained 115 neonatologists (median 8.5, range 0–18) and 499 pediatricians (median 28, range 10–100). Currently, 21 out of 22 NICUs (95.4%) offer structured training programs annually.

#### 4.4.2. Nursing Staff

Neonatal nursing staff in Greece may consist of a combination of nurses and midwives, midwives only, or nurses only (distributed, respectively, across 12, 5, and 5 NICUs in 2022). Nationwide, there were 506 nurses/midwives (median 18.5, range 10–57) working across 22 NICUs (21 Level III and 1 Level II).

In 2004, 611 nurses/midwives (median age 29.5, range 13–54) were employed across 20 NICUs, reflecting a significant 17.2% decrease in employment by 2022 (*p* = 0.034). In 2022, of the 506 nurses/midwives, a median of five (range: 1 to 9) worked during morning shifts, a median of three (range: 1 to 8) during afternoon shifts, and a median of three (range: 1 to 5) during night shifts (*p* = 0.001), indicating that the number of nurses/midwives differs among the shifts. 

A positive correlation was documented in 2022 between the number of nursing staff and the number of intensive care beds in each center (r_s_ = 0.672, *p* = 0.001), as well as between the number of nursing staff and NICU admissions (r_s_ = 0.539, *p* = 0.01).

### 4.5. Facilities and Technological Equipment

#### 4.5.1. Neonatal Care Beds and Incubators

Table 2 displays the absolute numbers of operational NICU beds in 2004 and 2022, their categorization by levels of neonatal care, and the ratios of NICU beds per 10,000 live births. Additionally, it provides data on the ratios of neonatologists and nursing staff to 10,000 live births and NICU beds and the ratios of neonatologists to LBW-VLBW infants.

Among the 22 NICUs in 2022, there were 632 incubators (median: 24.5, range: 9–68), including 60 open incubators, 537 closed incubators, and 35 hybrid incubators (capable of functioning both as open and closed systems). Of them, 134 (21.2%) were under 5 years old, 199 (31.5%) were 5–10 years old, and 299 (47.3%) were over 10 years old. Moreover, the number of incubators in each NICU was positively correlated with the number of admitted neonates (r_s_ = 0.491, *p* = 0.02). Data on the number and types of incubators were not available for 2004.

#### 4.5.2. Other Medical Devices

In 2022, across 20 Level III and 1 Level II NICUs, a total of 218 ventilators were available for mechanical ventilation (median: 10 per unit; range: 3–23), equating to 0.76 ventilators per intensive care bed. Of these, 112 ventilators were designated for Conventional Mechanical Ventilation (CMV), 4 for High-Frequency Oscillatory Ventilation (HFOV), and 75 for both CMV and HFOV. Additionally, 18 Level III NICUs and 1 Level II NICU utilized 119 devices for the application of nasal Continuous Positive Airway pressure (nCPAP) (median: 6; range: 1–22), with 0.42 devices per intensive care bed. In 2004, each NICU had a median of 10.5 (range 2–14) ventilators. Of the 188 ventilators (0.71 ventilators per intensive care bed), 12 could provide HFOV only, while 13 could provide both CMV and HFOV. No data were available regarding the nCPAP devices. Overall, between 2004 and 2022, while the number of ventilators per NICU remained relatively unchanged (*p* = 0.818), the ratio of ventilators to intensive care beds showed a significant increase (*p* < 0.001).

In 2022, the daily use of blood gas analyzers, X-rays, and ultrasound devices was standard across all 22 NICUs, with each unit equipped with transducers for brain and cardiac ultrasounds, and 18 out of 22 having lung ultrasound capabilities. In 2004, 16/20 NICUs had an ultrasound device (80%). In 2022, brain imaging via magnetic resonance imaging (MRI) was available for high-risk neonates in 17 of the 22 NICUs (77.3%). Moreover, hearing assessments were performed in 20 of the 22 NICUs (90.9%) using otoacoustic emissions and, in 16 of these 20 NICUs (72.7%), using auditory evoked potentials. Also, modern monitoring tools included Near-Infrared Spectroscopy (NIRS), which was available in 5 NICUs, transcutaneous CO_2_ monitoring (TcCO_2_), which was available in 1 NICU, amplitude-integrated EEG (aEEG) or EEG, which were available in 13 NICUs, and continuous blood pressure monitoring, which was available in 6 NICUs. The latter information was not available for 2004.

Based on the available data for 2022 only, in the delivery rooms across 19 NICUs, 18 (94.7%) utilized pulse oximeters and NeoPuff resuscitators, 17 (89.5%) had oxygen blenders, and 11 (57.9%) used ECG devices during neonatal resuscitation.

### 4.6. Therapeutic Interventions and Access to Specialized Treatments

In 2022, 16 out of the 21 (76.2%) level III NICUs reported using devices for administering inhaled Nitric Oxide (iNO). Additionally, seven NICUs (one in Athens, three in Thessaloniki, one in Ioannina, one in Patras, and one in Heraklion) had the capability to provide therapeutic hypothermia for asphyxiated newborns. In contrast, in 2004, only 25% of the participating centers used iNO, and therapeutic hypothermia was not practiced. At both time points of the survey, no NICU in Greece offered Extracorporeal Membrane Oxygenation (ECMO) therapy for life-threatening cardiorespiratory failure. In 2022, 12 out of 22 Level III NICUs were affiliated with pediatric surgery departments, while only 4 (all located in Athens) were connected to pediatric cardiac surgery departments.

Total parenteral nutrition solutions were primarily prepared within the NICUs in 12 of the centers (11 in 2004), in pharmacy departments in 5 (down from 9 in 2004), in hospital parenteral departments in 2, and by a sponsoring company in 1. According to the 2022 registry, for parenteral solution preparation, a pre-specified program was used in 13 out of 21 NICUs (61.9%), while in 19 out of 21 NICUs (90.5%), the formula was calculated by a physician.

Lastly, 12 out of 22 NICUs (54.5%) reported conducting surgical operations at supporting hospitals. Laser photocoagulation for the Retinopathy of prematurity was done in 8 out of 22 NICUs, and central venous catheter insertion was performed in 17 out of 22 NICUs. No relevant data were available for 2004.

### 4.7. Neonatal Follow-Up Programs

In 2022, 20 out of 22 NICUs reported offering long-term monitoring and follow-up programs. The expert teams involved in these follow-ups primarily consisted of neonatologists (18 out of 20), physiotherapists (12 out of 20), ophthalmologists (9 out of 20), pediatric neurologists (8 out of 20), orthopedic specialists (4 out of 20), psychologists (4 out of 20), and speech therapists (3 out of 20). In 2004, 17 NICUs reported having an organized long-term follow-up program comprising similar medical specialists.

Specific details on the duration of the follow-up programs were only available for 2022, with 17 NICUs reporting varying timeframes: 2 NICUs had a maximum duration of 3 months, 1 NICU followed up for 12 months, 9 NICUs for 24 months, 1 NICU for 30 months, 3 NICUs for 3 to 4 years, and 1 NICU for more than 4 years. The primary tools used for neurodevelopmental assessments, covering areas such as gross motor skills, fine motor skills, cognition, language, and behavior, include the Bayley-III test (four NICUs); the Denver test (one NICU); the Griffiths test (one NICU); the Modified Checklist for Autism in Toddlers, Revised (M-CHAT-R) (one NICU); and a combination of the Bayley-III test with another assessment tool (four NICUs).

### 4.8. NICU Affiliations and Services

The 2022 registry provided additional insights into NICU structure and functioning, which were previously unrecorded. Most NICUs (15 out of 22, or 68.1%) were part of larger perinatal centers and affiliated with level III Obstetric Clinics (19 out of 22, or 86.3%). Moreover, they had departments for normal newborns (19 out of 22 NICUs, or 86.3%), collaborated with various subspecialties (17 out of 22 NICUs, or 77.2%), and all offered Rooming-in options. In terms of family presence, 19 out of 22 NICUs allowed visits during designated hours, while 3 offered unrestricted visiting hours. Parental accommodation was available in 8 of the 22 NICUs, with only 1 NICU providing an individual ward for each sick newborn. Additionally, all hospitals had a Nosocomial Infections Committee and a Pharmacy Service, and a substantial proportion reported having a Bioethics Committee (63.6%).

## 5. Discussion

This questionnaire-based survey analyzed the care provided to sick neonates in Greek NICUs, examining their organization and operation alongside national perinatal health data, and comparing two time periods nearly 20 years apart. From 2004 to 2022, there has been an increase in the number of NICUs, available beds, admission rates, birth coverage, and medical staff, even though nursing staff numbers have declined. Additionally, NICU equipment has improved, and new therapeutic interventions have become available. Moreover, most NICUs reported offering long-term follow-up programs in 2022. Despite these advancements in neonatal care infrastructure, birth rates have decreased significantly, while prematurity rates have risen.

Developed countries offer specialized perinatal and neonatal intensive care for high-risk newborns and pregnancies through organized networks and regionalized systems [15]. In the United States, the American Academy of Pediatrics has defined the levels of neonatal care to standardize services across healthcare facilities [9,14]. Moreover, the levels and distribution of NICUs across regions influence the workforce and technological resources needed in each country; higher-acuity NICUs, such as Level III and IV units, require more specialized personnel and more advanced equipment compared to lower-level units [11].

According to this survey, the number of Level II/III NICUs in Greece’s seven healthcare regions has slightly increased from 2004 to 2022, with 81.5% participating in the 2022 registry. The NICU capacity, reflected in the increase in operational beds and admissions, has also grown, despite the socioeconomic challenges of the past decade. Nevertheless, NICU expansion has been uneven across regions, with areas of higher population density experiencing more growth. This discrepancy largely reflects Greece’s geographical and demographic landscape, as most of the population is concentrated in its two largest cities, leading to a higher incidence of prematurity, especially in Athens. Notably, no NICU in the country meets the criteria for Level IV designation, which involves providing the highest level of care for critically ill and premature newborns, including the use of ECMO for severe cardiorespiratory failure (e.g., in infants with congenital diaphragmatic hernia or meconium aspiration syndrome) [9]. Furthermore, all the operational cardiac surgery centers are in Athens, raising concerns about the transportation of infants with critical congenital heart disease to these facilities. It is anticipated that a new pediatric hospital with a cardiac surgery center will soon be established in Thessaloniki, Greece’s second-largest city. Other studies have also highlighted a misalignment between the distribution of NICU resources and regional needs for advanced care, raising concerns about equity, efficiency, and the potential impact on healthcare outcomes and costs [16].

Previous studies have reported various NICU per 10,000 live births ratios across North America, Australia, and Europe, ranging from 2.92 in the United Kingdom to 0.66 in Paris, France [17,18]. In our study, we observed an increase in Level III NICUs per 10,000 live births from 2.1 in 2004 to 3.16 in 2022. This change, although not statistically significant, may indicate an enhancement in neonatal care infrastructure over this period, potentially in response to increasing healthcare needs, improvements in neonatal care, or changes in birth rates that have affected NICU availability relative to the number of live births. However, notable disparities still existed in 2022 among the Regional Health Authorities regarding the distribution of Level II/III NICUs per 10,000 live births.

The data from the present survey showed that NICU admissions increased by 16.1% from 2004 to 2022, and the rate of NICU-admitted neonates per live birth rose by 61.5%, even though live births significantly declined during the study period. A similar trend has been observed in the United States, where NICU admission rates increased by 37% from 2008 to 2018, irrespective of the racial and ethnic groups [19]. The higher prematurity rate could be another explanation for the increased admissions. Since 1980, preterm birth rates in Greece have risen significantly and steadily, although they have plateaued since 2009 [12]. Preterm birth rates have been reported to increase in some high-income countries, while, globally, the majority of preterm births occur in the late preterm period [20]. The reduction of non-medically indicated cesarian sections and delivery by induction, as well as the limitation of multiple embryo transfers in assisted reproduction are among the proposed interventions to reduce prematurity rates in the United States by 2030 [21]. Another possible reason could be that in Greece, active resuscitation is now provided from 22 weeks of gestation. Additionally, the increasing maternal age at birth over time [29.7 years in 2004 and 32 years in 2021 (latest available data)], possibly associated with higher maternal morbidity, may have influenced neonatal admissions. Interestingly, the majority of NICU admissions in both time periods involved infants born at term. This finding could be attributed to the insufficient number of Level II NICUs in the country, which leaves term, less critically ill infants with no option but to receive medical care in higher-level NICUs. Despite the increase in total admissions, however, we observed that the median number of admissions per NICU decreased. This observation suggests potential changes in the distribution of admissions across NICUs, variations in the NICUs’ capacity, or shifts in regional healthcare utilization patterns over time. Overall, the rise in NICU admissions likely reflects improved access to specialized care for high-risk infants, driven by expanded healthcare coverage and increased NICU availability [19], suggesting that the availability of services may influences their utilization.

Previous studies have shown that high-level NICUs, which manage a higher volume of admissions, are associated with lower odds of neonatal mortality compared to lower-level NICUs with fewer admissions [22]. Similarly, a recent French investigation involving preterm infants born between 24 and 30 weeks of gestation reported lower survival rates in low-volume Level III NICUs; however, there was no increase in disabilities at 2 years [23]. In our survey, admission volumes varied widely among the participating NICUs. However, since we did not investigate the infants’ outcomes, no conclusions could be drawn about the impact of NICU capacity (the number of admissions) on survival. Encouragingly, most premature infants under 32 weeks’ gestational age in this study were born in hospitals with NICU support. This aligns with advances in NICU and perinatal care and supports fetal in utero transfer policies, which help ensure better outcomes [24].

Available medical and nursing personnel, alongside technological infrastructure, are critical determinants of the level of care provided in the NICU setting [9]. Moreover, the clinical demands on NICUs, as seen in the growing number of admitted neonates and improved survival rates for extremely premature infants highlight the need for a larger and better-trained workforce [25]. In line with this, the number of neonatologists has been found to be independently associated with variations in mortality rates among very premature infants across different NICUs [26]. Our findings show that board-certified neonatologists in Level II/III NICUs rose (although not significantly) by 21.8% between 2004 and 2022. The ratio of neonatologists per 10,000 live births also increased significantly in our country from 14.8 in 2004 to 25 in 2022. Earlier studies published around 2004 reported lower ratios, ranging from 2.7 in the United Kingdom to 6.1 in the United States [17]. A recent study from the United States reported that between 1991 and 2017, the number of neonatologists per 10,000 live births increased by 200%, rising from 4 to 14 neonatologists per 10,000 live births [16]. In a survey from Korea involving 87 hospitals that operated NICUs, 2.1 and 3 neonatologists per 10,000 live births were reported in 2009 and 2015 [27].

Nevertheless, despite an increase in the Greek NICU medical workforce, the number of physicians trained in neonatology in the country has declined in recent years. This trend may stem from the challenging NICU environment, the complexity of cases, and the increasing non-clinical responsibilities (teaching, administrative duties, etc.) alongside personal factors that contribute to higher turnover rates and burnout [28].

We also observed a significant shortage of nursing personnel, which has notably worsened over the past two decades, despite an increase in the number of NICU beds. It should be noted that nurses in Greece work in three shifts, with a significant imbalance, particularly during night shifts. According to our findings, the nursing staff ratio is roughly one-half to one-third of that observed in other countries [27,29]. The European Standards of Care recommend the following nurse-to-infant ratios: 1:1 for intensive care, 1:2 for intermediate care, and 1:4 for special care [30]. Research indicates possibly improved neonatal outcomes with higher nurse-to-infant ratios [31], while NICU nurse understaffing has been associated with an increased risk for VLBW nosocomial infection [32]. Nevertheless, we did not evaluate nursing staff in relation to the acuity of the infants. In a survey (2008) from the United States, the patient-to-nurse ratio ranged from 2.78 for the lowest-acuity infants (continuous care) to 1.04 for the highest-acuity infants (unstable/complex critical care) [29]. This research highlights significant challenges in maintaining a high level of nursing care and managing the associated workload in Greek NICUs, an issue that requires serious attention from the national Ministry of Health. Increasing nurse-to-infant ratios could create a more efficient healthcare model of neonatal care and help address potential staffing shortages in the future.

NICUs provide a range of care, from basic to complex and specialized treatments, employing advanced technology. Neonatal care has made remarkable progress since the 1960s, particularly in respiratory support for critically ill preterm and term infants [3], as well as in other areas like the management of hypoxic-ischemic encephalopathy through therapeutic brain cooling [33]. According to the survey results, the medical resources in Greek NICUs, including devices used in neonatal care, appear to be satisfactory overall. Standard NICU equipment and newer treatment modalities, such as therapeutic hypothermia, have been introduced, along with various monitoring devices. However, at the regional level, the distribution of NICU medical equipment is often uneven. For instance, the presence of only one hypothermia device in the entire Athens district, which accounts for nearly half of the nation’s births, underscores the issue of maldistribution and the potential medico-legal implications of failing to provide the standard of care [34].

The 2022 NICU registry data highlights significant areas for improvement in neonatal care and family support. The high integration of NICUs within larger perinatal centers and their affiliation with Obstetric Clinics suggests a coordinated approach to maternal and neonatal care Additionally, the availability of Rooming-in options across all the NICUs underscores a commitment to family-centered care, promoting early bonding and potentially improving outcomes. However, only a few NICUs offer unrestricted visiting hours, and less than half of the participating centers offer parental accommodation, which could place strain on families and affect their ability to participate actively in care routines. Lastly, the presence of support services, such as pharmacy and Nosocomial Infections Committees in all the hospitals, along with Bioethics Committees in 63.6% of the NICUs, signifies essential strides toward providing high-quality care for both sick infants and their families.

Neonatal follow-up is crucial for monitoring and supporting the long-term health and development of infants, especially those born prematurely, with a low birth weight, or who require intensive care [35]. The majority of the participating NICUs reported offering long-term monitoring and follow-up programs in 2022, primarily led by neonatologists. However, the specific follow-up durations varied across the NICUs, and several neurodevelopmental assessment tools were used. This finding aligns with a recent study conducted across 11 European countries, which reported significant variation in follow-up policies and programs for children born very prematurely [36].

This survey is the first to present data on the organization of NICUs in Greece and, more generally, neonatal care, highlighting both the progress made over time and the significant operational gaps that still exist. Several limitations of this study should be acknowledged. First, the survey relied on questionnaires, which come with inherent challenges, such as potential issues with data accuracy and quality, ambiguous questions, and incomplete responses. Furthermore, one could argue that the use of these questionnaires did not capture all the annual live births and, most importantly, preterm infants, compared to the centralized data from the national statistical authority (55.5% and 73.3% coverage, respectively, for 2022). This limitation is indeed valid and can be attributed to the fact that this survey did not include all the hospitals where births take place, as well as the non-participation of some of the NICUs in the study. Nonetheless, due to the absence of a centralized national registry containing information about NICUs and the care they provide, questionnaires were the only feasible method to depict the organization and functioning of NICUs across the country. Despite these challenges, the high response rate in our study, which covered key areas, enhances the reliability and significance of our findings. Additionally, the study did not address some important issues, such as the specific reasons for admissions to NICU, disease acuity or the evaluation of the efficiency of NICU-provided healthcare, and the associated workload. There is also a notable gap in the data regarding the long-term outcomes of NICU care and the impact of regional disparities on healthcare access. Addressing these gaps in future research would provide a more holistic understanding of NICU operations and outcomes nationwide.

## 6. Recommendations

Based on the results of our national survey, the proposals for improving neonatal intensive care include the following:

- The development of a central registry database to monitor NICU operations nationwide, ensuring standardized data collection and facilitating decision making for policy and care improvements. Data on the association between NICU capacity, infrastructure, and neonatal outcomes should be analyzed on a national and regional basis and compared with international standards.
- NICUs function within a regionalized healthcare system. It is essential to develop more intermediate (Level II) NICUs, minimizing unnecessary admissions to higher-level NICUs and optimizing care allocation.
- The implementation of sustainable policies to recruit and retain neonatologists, addressing the decreasing number of physicians trained in neonatology. Incentives and professional development opportunities to attract and retain specialists should be developed.
- The implementation of measures to train, recruit, and retain skilled neonatal nurses, addressing the significant shortage of nursing staff with regard to the international standards for nurse-to-neonate ratios.
- Developing a national guided training program for physicians and nurses could help create a highly qualified workforce, thereby ensuring an improved quality of care.
- Infrastructure renewal must align with the most recent advancements in neonatal therapies.
- Communicate the study results to the Ministry of Health and advocate for increased resources through relevant bodies, such as the Hellenic Neonatology Society. The associations of the parents of premature newborns and the media can play a significant role in supporting these efforts.

## 7. Conclusions

This survey highlights significant changes in Greek NICUs and perinatal health data over nearly two decades (2004–2022). We documented an expansion in NICUs and resources, with growth in the number of NICUs, beds, and available medical staff, indicating an enhanced capacity to admit and care for sick infants. However, a decrease in nursing staff poses potential challenges to maintaining the standards of neonatal care. The equipment in NICUs has improved, and new therapeutic interventions have been introduced, reflecting advancements in the quality and capacity of neonatal care.

Despite these improvements in neonatal care infrastructure, Greece has experienced a decline in birth rates alongside an increase in prematurity rates. These trends may reflect shifting population dynamics and emerging health challenges in maternal and infant health.

Overall, the findings suggest that while NICU infrastructure and care capabilities have advanced, the health system faces demographic shifts and staffing challenges that could impact neonatal outcomes in the long term.

## Figures and Tables

**Table 1:** The distribution of NICUs by Regional Health Authorities (RHAs) and the respective geographical regions, as well as the levels of care in relation to live births, prematurity rates, and the number of NICUs per 10,000 live births for the year 2022.

RHA	Geographical Region	NICUs (*n*, %)	Level III (*n*)	Level II (*n*)	Live Births (*n*, % of Annual Live Births) *	Prematurity Rate (%) *	NICUs/10,000 Live Births
1	Attica	10 (37)	8	2	28,125 (37)	13.4	3.55
2	Piraeus and North Aegean + South Aegean	3 (11.1)	3	0	4518 (5.95)	10.53	6.64
3	Central and West Macedonia	1 (3.7)	1	0	13,725 (18)	9.4	0.72
4	Eastern Macedonia and Thrace	5 (18.5)	5	0	3865 (5.09)	12.2	12.93
5	Thessaly and Central Greece	2 (7.4)	2	0	7927 (10.44)	11.39	2.52
6	Epirus, Ionian Islands, West Greece, and Peloponesse	3 (11.2)	3	0	11,989 (15.79)	12.15	2.5
7	Crete	3 (11.1)	2	1	5772 (7.6)	11.01	5.19

* Data derived from the Hellenic Statistical Authority (ELSTAT).

**Table 2:** Trends in NICU beds, staffing, and care ratios from 2004 to 2022.

Year and Number of Participating NICUs (*n*)	2004 (20)	2022 (22)	Change (%)	*p*
NICU beds (*n*)	903	959	+6.2	N/A
Intensive care beds (*n*, %)	264 (29.2)	284 (29.6)	+7.5	N/A
Intermediate care beds (*n*, %)	317 (35.1)	234 (24.4)	−26.2	N/A
Beds for care before discharge (*n*, %)	322 (35.6)	384 (40)	+19.2	N/A
Isolation beds (*n*, %)	Not recorded	57 (5.94)	N/A	N/A
NICU beds/10,000 live births	85.6	126.3	+46.9	<0.001
Neonatologists/NICU bed	0.17	0.12	+16.5%	0.002
Neonatologists/Intensive care bed	0.59	0.66	+11.8	<0.001
Neonatologists/10,000 live births	14.8	25	+69	<0.001
Neonatologists/1000 low-birth-weight infants	17	25	+47	<0.001
Neonatologists/1000 very-low-birth-weight infants	140.7	225.6	+60.38	<0.001
Nursing staff/10,000 live births	57.9	66.6	+15	0.019
Nursing staff/NICU bed	0.67	0.52	−22.4	<0.001
Nursing staff/Intensive care bed	2.31	1.78	−22.9	<0.001

Not applicable (N/A): Data did not permit meaningful calculation or statistical testing.

## Data Availability

The original contributions presented in the study are included in the article/Supplementary Material, further inquiries can be directed to the corresponding author.

## Supplementary Materials

The following supporting information can be downloaded at: https://www.mdpi.com/article/10.3390/children12010085/s1, File S1: Survey questionnaire.
