# Supplementary material for: A Survey on the Organization and Operation of Level II/III Neonatal Intensive Care Units in Greece: A Comparison Between 2004 and 2022
Source: Children (Basel). 2025 Jan 13;12(1):85. doi: 10.3390/children12010085 (PMC11764378; doi:10.3390/children12010085)
Supplement: Supplementary file 1 [file children-12-00085-s001.zip › children-3412636-supplementary.pdf]

QUESTIONNAIRE ON THE ORGANIZATION AND OPERATION OF LEVEL II/III NEONATAL INTENSIVE CARE UNITS (NICUs) IN GREECE

MARCH 2023

1. NICU name participating in the survey:
……………………………………………………………………………………………………

2. The NICU is:
- ☐ Private
- ☐ Public

3. If it is public, please specify which entity it belongs to:
- ☐ NHS
- ☐ University

4. It belongs to a:
- ☐ General Hospital
- ☐ Maternity Hospital
- ☐ Pediatric Hospital
- ☐ Other

5. It is:
- ☐ An autonomous clinic
- ☐ Pediatric Department
- ☐ Other

If you selected "Other" in the previous item, please specify:
……………………………………………………………………………………………………

6. It belongs to the following Regional Health Authority:
- ☐ 1st
- ☐ 2nd
- ☐ 3rd
- ☐ 4th
- ☐ 5th
- ☐ 6th
- ☐ 7th

7. Prefecture:
……………………………………………………………………………………………………

8. Geographic region
……………………………………………………………………………………………………

9. Year the NICU began providing services:
……………………………………………………………………………………………………

Level of NICU and capacity

10. NICU level: .
- ☐ Level of Care I
- ☐ Level of Care I & II
- ☐ Level of Care I, II & III

11. The number of infants that can be hospitalized in the NICU:
……………………………………………………………………………………………………

12. The number of infants that can be hospitalized in an intermediate care facility:
……………………………………………………………………………………………………

13. The number of infants that can be accommodated in an incubator for growth:
……………………………………………………………………………………………………

14. The number of infants that can be accommodated in cribs in the NICU before being discharged.
……………………………………………………………………………………………………

15. The number of infants that can be accommodated in an isolation room
……………………………………………………………………………………………………

Operation Record during 2022

16. Total number of NICU admissions during 2022:
…………………………………………………………………………………………
17. The number of inborn infants among the total admissions.
…………………………………………………………………………………………
18. The number of hospital admissions for infants born at less than 37 weeks of gestation:
…………………………………………………………………………………………
19. The number of hospital admissions for infants born between 34 weeks and 36 weeks + 6 days of gestation:
…………………………………………………………………………………………
20. The number of hospital admissions for infants born at less than 32 weeks of gestation:
…………………………………………………………………………………………
21. The number of inborn infants born at less than 32 weeks of gestation among the total NICU admissions.…………………………………………………………………………………………
22. The number of NICU admissions for infants born at less than 28 weeks of gestation:
…………………………………………………………………………………………
23. The number of inborn infants born at less than 32 weeks of gestation among the total NICU admissions:
…………………………………………………………………………………………
24. The number of admissions for infants with a birth weight $\geq$ than 2500 grams:
…………………………………………………………………………………………
25. The number of admissions for infants with a birth weight less than 2500 grams:
…………………………………………………………………………………………
26. The number of admissions for infants with a birth weight less than 1500 grams:
…………………………………………………………………………………………
27. The number of admissions for infants with a birth weight less than 1000 grams:
…………………………………………………………………………………………
28. Is the NICU part of a Perinatal Center?
    - ☐ YES
    - ☐ NO
29. Does the hospital where the NICU operates have a Maternity Ward?
    - ☐ YES
    - ☐ NO
30. Does the hospital have pediatric subspecialties?
    - ☐ YES
    - ☐ NO
31. If there are pediatric subspecialties or clinics with doctors specializing in infant care, please select the applicable ones.
    - ☐ Radiology
    - ☐ Paediatric Surgery
    - ☐ Cardiac Surgery
    - ☐ Ophthalmoly
    - ☐ Cardiology
    - ☐ Neurosurgery
    - ☐ Orthopedics
    - ☐ Otolaryngology
    - ☐ Dermatology
    - ☐ Pediatric Infectious Diseases
    - ☐ Pediatric Neurology
    - ☐ Genetics
    - ☐ Other: ………………………………………………………………………………
32. Does your hospital have an Infections Committee?

☐ YES
☐ NO

33. Does your hospital have a Bioethics Committee?
☐ YES
☐ NO

34. Does your hospital have a Quality Department?
☐ YES
☐ NO

35. Does your hospital have a Pharmacy Service?
☐ YES
☐ NO

36. Does your hospital have a unit for full-term infants?
☐ YES
☐ NO

37. Is rooming-in practiced in the full-term maternity ward?
☐ YES
☐ NO

38. Does your hospital have a Human Milk Bank?
☐ YES
☐ NO

NICU Equipment

39. The total number of incubators available:
……………………………………………………………………………………

40. The total number of open incubators available:
……………………………………………………………………………………

41. Total number of closed incubators available:
……………………………………………………………………………………

42. Total number of hybrid incubators available:
……………………………………………………………………………………

43. Is there a neonatal transport incubator equipped with a ventilator?
☐ YES
☐ NO

44. The number of incubators produced in the last five years:
……………………………………………………………………………………

45. The number of incubators that are 5 to 10 years old:
……………………………………………………………………………………

46. The number of incubators that are over 20 years old:
……………………………………………………………………………………

47. The total number of ventilators:
……………………………………………………………………………………

48. The number of ventilators designated exclusively for infant use:
……………………………………………………………………………………

49. The number of ventilators capable of being used for infants:
……………………………………………………………………………………

50. The number of ventilators used exclusively for conventional ventilation:
……………………………………………………………………………………

51. The number of ventilators used exclusively for high frequency ventilation:
……………………………………………………………………………………

52. The number of ventilators that provide both high-frequency and conventional ventilation modes:
……………………………………………………………………………………

53. The number of CPAP (Continuous Positive Airway Pressure) devices:
……………………………………………………………………………………………

54. The number of iNO (inhaled Nitric Oxide) devices:
……………………………………………………………………………………………

55. Is Therapeutic Hypothermia available in the NICU?

☐ YES

☐ NO

56. Is it possible to administer iNO (inhaled Nitric Oxide)?

☐ YES

☐ NO

57. Is aEEG (amplitude-integrated EEG) monitoring available?

☐ YES

☐ NO

58. Is there a capability for invasive blood pressure measurement?

☐ YES

☐ NO

59. Is pulse oximetry available?

☐ YES

☐ NO

60. Is transcutaneous monitoring (tcCO2) available?

☐ YES

☐ NO

61. Is cerebral oximetry (NIRS) available?

☐ YES

☐ NO

62. Is ultrasound imaging available for the brain or other body organs?

☐ YES

☐ NO

63. Are otoacoustic emission (OAE) tests available?

☐ YES

☐ NO

64. Are evoked potential tests available?

☐ YES

☐ NO

65. Are portable x-ray machines available?

☐ YES

☐ NO

66. Is MRI imaging available for the brain or other body organs?

☐ YES

☐ NO

67. Is there an isolation room with negative air pressure available for infected infants?
- ☐ YES
- ☐ NO

In the same hospital, is it possible to perform:

68. Major pediatric surgery?
- ☐ YES
- ☐ NO

69. Surgical placement of central venous catheters?
- ☐ YES
- ☐ NO

70. Laser therapy for ROP?
- ☐ YES
- ☐ NO

71. Cardiothoracic surgery?
- ☐ YES
- ☐ NO

72. Other surgical interventions (please describe).
……………………………………………………………………………………………………

Total Parenteral Nutrition

73. Dedicated area for preparing formulas or solutions for parenteral nutrition:
Please, select only one option.
- ☐ In the NICU
- ☐ At the pharmacy
- ☐ Other: ……………………………………………

74. If you selected "Other" in the previous item, please specify the location:

……………………………………………………………………………………………………

75. A special program is used to prepare Total Parenteral Nutrition formulas, with compositions individualized based on the needs of each infant.
- ☐ YES
- ☐ NO

76. The composition of parenteral feeds is individualized for each infant and evaluated by the doctor rather than using standardized software.
- ☐ YES
- ☐ NO

77. Standardized commercial parenteral nutrition bags are used.
- ☐ YES
- ☐ NO

Other areas in NICU

78. s there a mini laboratory available?
- ☐ YES

☐ NO

79. Is there a blood gas analyzer available?

☐ YES

☐ NO

80. Is there a bilirubinometer available?

☐ YES

☐ NO

81. Is there adequate storage space available?

☐ YES

☐ NO

82. Is there sufficient space for waste management?

☐ YES

☐ NO

83. Is there designated space for hand washing before entering the NICU?

☐ YES

☐ NO

84. Is there a changing room available for the medical staff in the NICU?

☐ YES

☐ NO

85. Is there a changing room available for the nursing staff in the NICU?

☐ YES

☐ NO

86. Is there a central heating and ventilation system in place?

☐ YES

☐ NO

87. Is the specialist doctor's on-call room located inside the NICU?

☐ YES

☐ NO

88. Is the intern's/resident's on-call room located inside the NICU?

☐ YES

☐ NO

89. Are there sufficient rooms available for staff education and training?

☐ YES

☐ NO

90. Are there sufficient offices available for the staff?

☐ YES

☐ NO

Please provide the number of offices that are available:
……………………………………………………………………………………………

91. Has the NICU undergone extensive renovations recently?

- [ ] YES
- [ ] NO

92. If yes, please specify the year in which they were completed:

....

NICU construction plan for family needs

93. What are the visiting hours for the NICU?

- [ ] Access is allowed only during specific hours of operation
- [ ] 24/7 access

94. Is it possible for parents to stay at the hospital?

- [ ] YES
- [ ] NO

95. Is there a single room for each sick infants?

- [ ] YES
- [ ] NO

Transportation of infants

96. Does the hospital that the NICU belongs to have its own ambulance for transporting sick infants?

- [ ] YES
- [ ] NO

97. Is the hospital with the NICU served by an ambulance that transports sick infants and is operated by the National Emergency Aid Centre?

- [ ] YES
- [ ] NO

98. If infants are transported via other means, please describe the method used:

....

99. Are deliveries conducted at your hospital?

- [ ] YES
- [ ] NO

100. How many deliveries were conducted in your center in 2022?

....

101. The number of infant resuscitation tables in the delivery room.:

....

102. Is there a NeoPuff resuscitator in the delivery room?

- [ ] YES
- [ ] NO

103. Does the transport incubator used in the delivery room have a ventilator?

- [ ] YES
- [ ] NO

104. Is there a pulse oximeter in the delivery room?

- [ ] YES
- [ ] NO

105. Is there a ECG Monitor in the delivery room?

- [ ] YES
- [ ] NO

106. Is there a gas blender in the delivery room?

- [ ] YES
- [ ] NO

What is the distance, in meters, between the delivery room and the NICU

....

Medical Staff (on the day the form is filled out)

107. The number of certified Neonatologists:

....
....

108. The number of certified Pediatricians:

....

109. The number of pediatricians pecializing in Neonatology:

....

110. The number of Pediatrics interns/residents:

....

Existing Medical Staff Positions

111. The number of certified Neonatologists:

....

112. The number of certified Pediatricians:

....

113. The number of Pediatricians specializing in Neonatology:

....

114. The number of Pediatric interns/residents:

....

Medical and nursing coverage of the NICU

115. Is the NICU staffed with medical personnel 24 hours a day?

- [ ] YES
- [ ] NO

116. Is there a doctor specializing in neonatology or a pediatrics intern/resident present alongside the neonatologist 24 hours a day in the NICU?

- [ ] YES
- [ ] NO

117. The number of midwives working in the delivery room:

....

118. The number of midwiferies in the NICU:

....

119. The number of nurses in the NICU:

....

120. The number of nursing staff working shifts in the NICU:

....

121. The number of nursing staff working in morning shift:

……………………………………………………………………………………………

122. The number of nursing staff working in afternoon shift:

……………………………………………………………………………………………

123. The number of nursing staff working in night shift:

……………………………………………………………………………………………

124. The nurse-to-infant ratio on the morning shift:

……………………………………………………………………………………………

125. The nurse-to-infant ratio on the afternoon shift:

……………………………………………………………………………………………

126. The nurse-to-infant ratio on the night shift:

……………………………………………………………………………………………

The NICU's clinical duties are supported by:

127. A speech & language therapist?

- [ ] YES
- [ ] NO

128. A psychiatrist?

- [ ] YES
- [ ] NO

129. A social worker?

- [ ] YES
- [ ] NO

130. A physiotherapist?

- [ ] YES
- [ ] NO

131. A psychologist?

- [ ] YES
- [ ] NO

132. A religious person?

- [ ] YES
- [ ] NO

Assistant staff

133. Is there a technology expert available for the mini laboratory?

- [ ] YES
- [ ] NO

134. Is there a ward assistant available?

- [ ] YES
- [ ] NO

135. Is there cleaning staff available?

- [ ] YES
- [ ] NO

Educational/Training activities in the NICU

136. Is there a specific annual educational/training syllabus for the NICU?

☐ YES

☐ NO

137. The number of resident/intern pediatricians who have been trained in the NICU over the last 5 years:

....

138. The number of pediatricians who have specialized in neonatology in your NICU over the last 5 years:

....

Long-term follow-up of high-risk infants

139. Do you have a follow-up clinic or department?

☐ YES

☐ NO

140. If YES, which of the following specialists participate in the team?

Please, select the specialties that apply.

☐ Neonatologists
☐ Pediatric Neurologists
☐ Ophthalmologists
☐ Orthopedics
☐ Physiotherapists
☐ Speech & Language Therapists
☐ Psychologists
☐ Other

If you selected "Other" in the previous item, please specify the specialty:

....
....

141. High-risk infants are followed up until the age of:

....

142. Is a psychomotor test (e.g., Bayley) used to assess the infants' skills?

☐ YES

☐ NO

143. If you selected "YES" in the previous item, please specify the type of psychomotor test used:

....

144. At what corrected age is the psychomotor test conducted? (Please provide the age in numbers):

....

Please write any comments or remarks you have about the present questionnaire:

....
....
....
....
....
....

....

....
